# Visualization of the biphasic calcium wave during fertilization in *Caenorhabditis elegans* using a genetically encoded calcium indicator

**DOI:** 10.1242/bio.059832

**Published:** 2023-08-21

**Authors:** Katie M. Toperzer, Savannah J. Brennan, David J. Carroll, Eric A. Guisbert, Karen S. Kim Guisbert

**Affiliations:** ^1^ Florida Institute of Technology, Biomedical Sciences Program, 150 W. University Blvd, Melbourne, FL 32901, USA; ^2^ Midwestern University, Department of Biochemistry and Molecular Genetics, 19555 N 59th Ave, Glendale, AZ 85308, USA

**Keywords:** Calcium, Fertilization, Genetically encoded calcium indicator, jGCaMP7s, Polyspermy, Oocyte-to-embryo transition

## Abstract

Fertilization is a critical step in development, yet internal fertilization events are notoriously difficult to visualize. Taking advantage of the calcium response that is a hallmark of sperm-egg fusion, we adapted the genetically encoded calcium indicator jGCaMP7s to visualize the moment of fertilization in *Caenorhabditis elegans* using fluorescence. We termed this tool the ‘CaFE’ reporter, for ‘calcium during fertilization in *C. elegans*’. The CaFE reporter produced a robust signal that recapitulated the previously reported, biphasic nature of the calcium wave and had no significant deleterious effects on worm physiology or fecundity. Calcium waves were not observed at the restrictive temperature in the *spe-9(hc88)* strain, in which sperm can still trigger meiotic maturation but can no longer fuse with the oocyte. Demonstrating the utility of the CaFE reporter, we analyzed polyspermy induced by inhibition of *egg-3* via RNAi and observed late calcium waves in the uterus. This finding provides support to the idea that calcium release is not restricted to the first sperm fusion event during polyspermy. Establishment of the CaFE reporter in the genetically tractable and optically transparent worm provides a powerful tool to dissect the oocyte-to-embryo transition inside a living animal.

## Teaser

**Summary:** We report the development of a genetically encoded calcium indicator in *C. elegans* to visualize the universally conserved calcium wave during fertilization.

## INTRODUCTION

The oocyte-to-embryo (OET) transition is a critical part of development that consists of three distinct phases in most organisms: meiotic maturation of the oocyte, fertilization and egg activation. Meiotic maturation entails release of the oocyte from cell cycle arrest in preparation for fertilization. Fertilization is the fusion between sperm and oocyte. Egg activation occurs immediately after fertilization and one or more blocks to polyspermy are enacted. Each of the separate stages of the OET have been examined in detail in multiple systems, particularly in those with external fertilization (e.g. sea urchins and *Xenopus*). In humans and other animals that have internal fertilization, these events are coordinated by extensive intercellular and intracellular signaling networks.

**Fig. 1.**
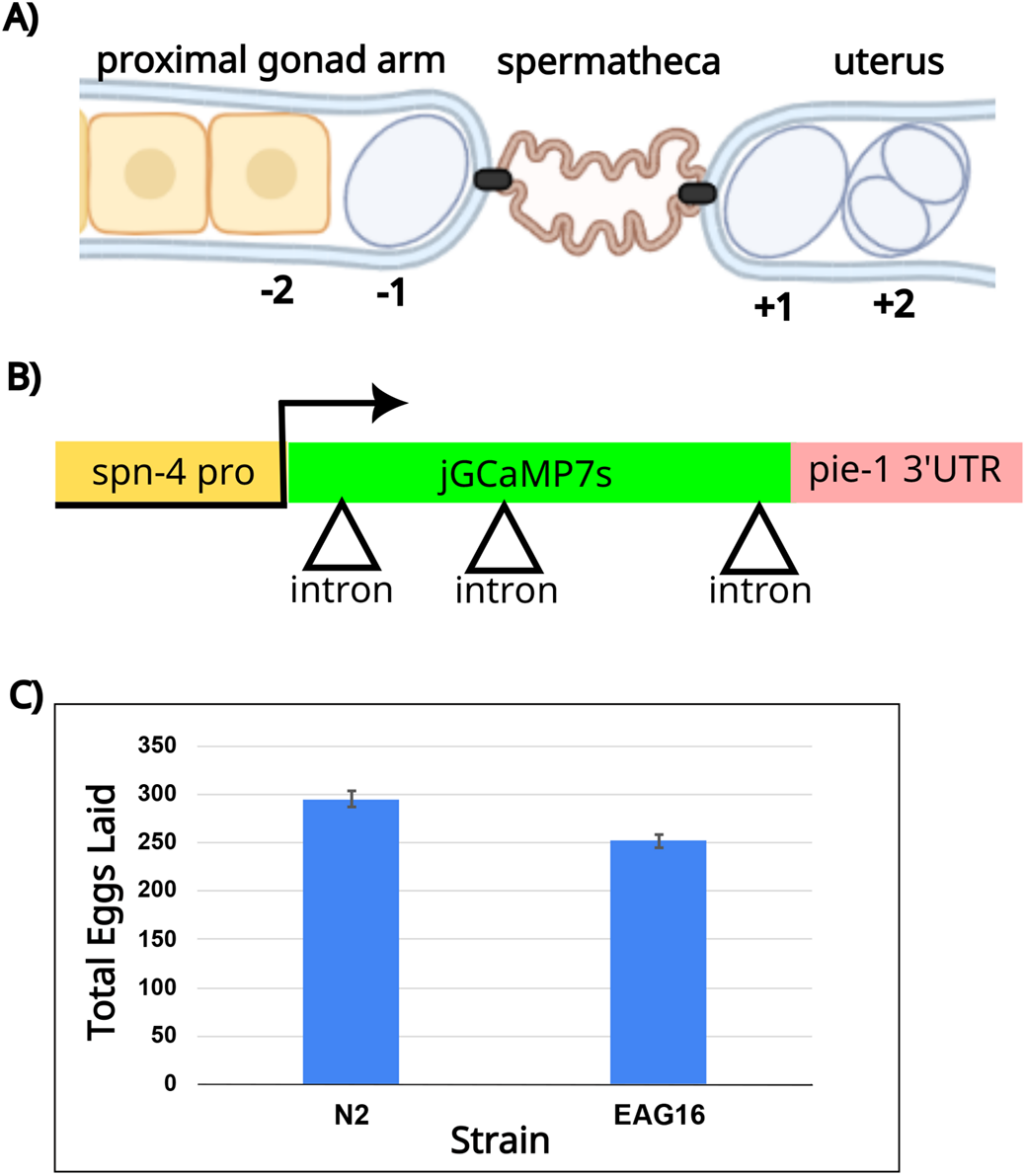
**Design strategy for a fertilization-specific GECI.** (A) Schematic of the worm gonad featuring a portion of the proximal gonad arm with a single-file row of developing oocytes, the spermatheca where the sperm are stored, and the uterus where embryos are stored temporarily before egg lay. The proximal oocyte has undergone meiotic maturation (shown by a change in shape and nuclear envelope breakdown) and is designated −1. The newly fertilized embryo in the uterus is labeled +1. Image created using Biorender.com. (B) Schematic of reporter design showing the *spn-4* promoter, the jGCaMP7s coding sequence interrupted by three introns (triangles) and the *pie-1* 3′ UTR. (C) Brood size analysis of the EAG16 reporter strain compared to N2 wildtype worms. Error bars indicate the s.e.m.

Internal fertilization events are notoriously difficult to visualize *in situ*. *Caenorhabditis elegans* has a unique advantage among model systems as it is optically transparent (Yamamoto et al., 2006). Furthermore, *C. elegans* has the unusual property of having a strictly ordered gonad, which allows a slice-through-time of the entire OET in a single image (Hubbard and Greenstein, 2000). The gonad consists of two symmetrical U-shaped tubes devoted to oogenesis connected to a spermatheca (storage organ for sperm in adult hermaphrodites), and a shared uterus and vulva (Fig. 1A). The single-file order of the oocytes and strict timing allows one and only one fertilization event at a time, approximately every 23 min per gonad arm. Its unique physiology, combined with powerful genetics and a wide array of existing cytological tools, makes *C. elegans* a compelling model in which to study the OET.

In *C. elegans*, many, but not all, phases of the OET are accompanied by morphological changes visible with standard Nomarski imaging. The proximal oocyte (the −1 oocyte) undergoes meiotic maturation in response to a secreted signal from the sperm. During meiotic maturation, the oocyte is released from a block in prophase I (diakinesis), the nucleus migrates to the distal end of the oocyte, and then the nuclear envelope breaks down. Cortical cytoskeletal rearrangements then separate the −1 oocyte away from the column of maturing oocytes, visibly changing the shape of the oocyte from columnar to ovoid. The distal constriction of the spermatheca then dilates and the oocyte is ovulated into the spermatheca, where it immediately becomes fertilized. After fertilization, meiosis completes, the male and female pronuclei meet, and the mitotic divisions follow, initiating embryogenesis. Each step of this OET is precisely orchestrated and relies on multiple signaling pathways between the sperm, oocyte and the gonadal sheath cells (Arur, 2017; Han et al., 2010; Hubbard and Greenstein, 2000; McCarter et al., 1997; Rose et al., 1997).

Unfortunately, the fertilization event remains difficult to visualize. The large size differential between the sperm and egg, often a difference of four or more orders of magnitude in volume in many species, increases imaging difficulty. Furthermore, sperm greatly outnumber and surround the oocyte. Even in *C. elegans,* in which hermaphrodites make a limited (∼300 total) sperm, correctly capturing the successful sperm fusion event is technically challenging.

The moment of fertilization, when a sperm fuses with an oocyte, is accompanied by a wave of intracellular calcium. The calcium wave is a conserved feature of fertilization in most species and is required to prevent polyspermy and to initiate embryogenesis (Stein et al., 2020). The calcium wave originates at the point of sperm entry and eventually encompasses the entire oocyte. Although the nature (timing and magnitude) of the wave can vary, every species so far examined displays a calcium signaling event during the OET (Stein et al., 2020).

Previously, this calcium wave has been detected by microinjection of calcium indicator dyes, such as Calcium Dextran Green or 1,2-bis(2-aminophenoxy)ethane-N,N,N′,N′-tetraacetic acid (BAPTA) derivatives, into oocytes. In worms, microinjection of calcium indicator dyes into the rachis of the worm gonad is required (Samuel et al., 2001; Takayama and Onami, 2016; Takayama et al., 2017). Although this method represented a tremendous advance in the field, this methodology is limited owing to its technical difficulty and low throughput.

Genetically encoded calcium indicators (GECIs) have been used with spectacular success by the neurobiology community. GECIs can instantaneously detect the transient surge of calcium coincident with the action potential. We chose the GCaMP series that were developed as part of the Genetically Encoded Neuronal Indicators and Effectors (GENIE) Project at Howard Hughes Medical Institute's Janelia Research Campus (Dana et al., 2019). The GCaMP design unites the calcium sensitivity of calmodulin with the *in vivo* fluorescence capability of GFP to create a sensitive reporter that glows only in the presence of calcium.

Here, we address the need for a fertilization reporter by co-opting the GECIs originally developed for neuronal imaging. The goal was to develop a system that can recapitulate the known dynamics of fertilization in order to more easily dissect the intricacies of the OET within a living organism.

## RESULTS AND DISCUSSION

### Design strategy

In order to develop a system to visualize the calcium wave of fertilization without microinjection, we co-opted the GECIs that have been used to study the calcium wave that occurs during neuronal signaling. As the calcium wave in fertilization is known to be transient, lasting on the order of seconds, the jGCaMP7s version was used as it was designed to have the most persistent signal (Dana et al., 2019). The sequence of jGCaMP7s was codon-optimized for expression in *C. elegans* and three artificial introns were inserted to promote expression (Fig. 1B) (Crane et al., 2019; Le Hir et al., 2003; Mello and Fire, 1995).

In order to capture fertilization events, the reporter was expressed in the mature oocyte. As germline expression in *C. elegans* is largely driven by 3′ untranslated regions (UTRs), the modified jGCaMP7s was inserted behind the *spn-4* promoter and in front of the *pie-1* 3′ UTR, which were previously reported to have maximal expression in the mature oocyte (Merritt et al., 2008). Furthermore, CRISPR was used to integrate the reporter in a single copy to avoid transgene silencing of multi-copy genes in the germline (Kelly et al., 1997). We integrated it into a safe harbor locus with an engineered split-hygromycin landing pad (strain PX696), which reconstitutes hygromycin resistance upon correct integration (Stevenson et al., 2020). The final strain (EAG16) was PCR-verified for integration into the correct genomic location and confirmed to be hygromycin resistant. The reporter strain displayed normal physiology and movement. The strain did not have a noticeable developmental delay but did display a slight decrease in overall brood size in comparison to the N2 wildtype strain (Fig. 1C). We termed this reporter system ‘CaFE’ for ‘calcium during fertilization in *C. elegans*’.

### The calcium wave is biphasic in *C. elegans*

**Fig. 2.**
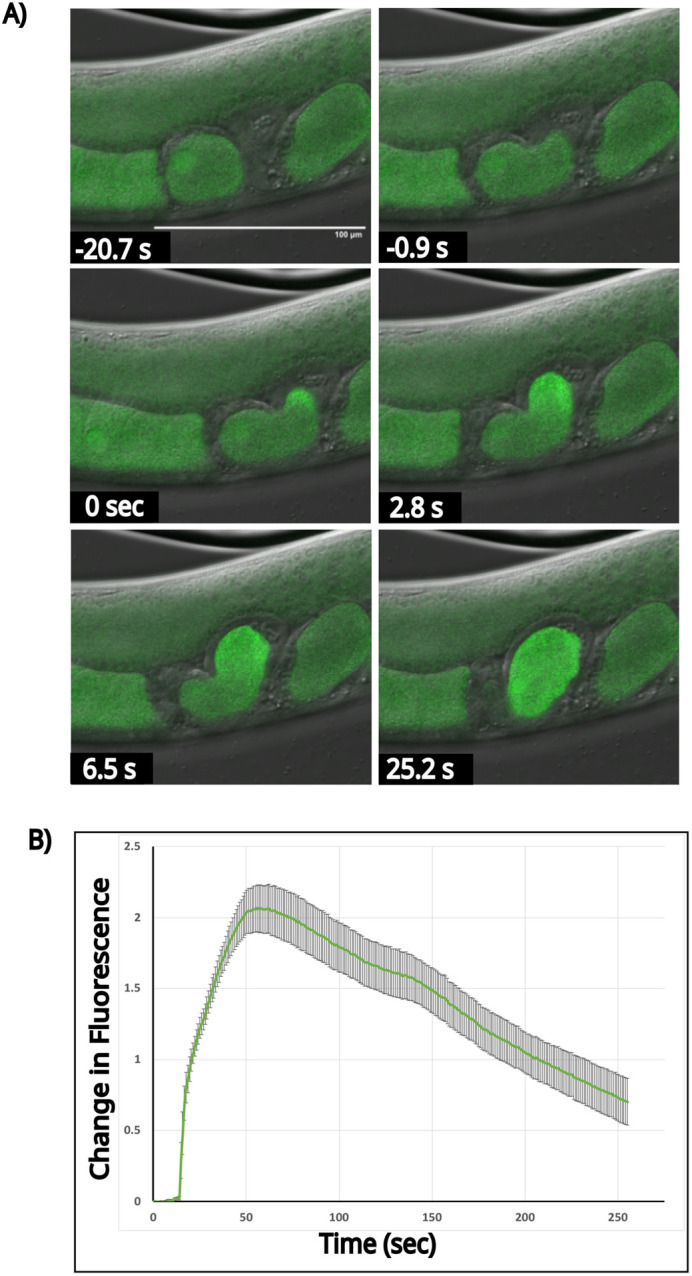
**Visualization of the biphasic nature of the calcium wave during fertilization.** (A) Time-lapse images of the calcium wave during fertilization. Time 0 was taken as the first frame showing a visible increase in fluorescence in the oocyte. Scale bar: 100 µm. (B) Quantitation of the change in fluorescence [(*F*_1_-*F*_0_)/*F*_0_)] over a region of interest encompassing the path of the oocyte during ovulation for a representative fertilization event shown above. The *x*-axis is time in seconds. The average intensity was plotted. Standard deviation is shown. Signals were normalized to the 15th frame before fertilization. *n*=7.

Taking advantage of the optically transparent nature of *C. elegans* along with its strictly ordered gonad, fertilization events in the CaFE reporter strain were visualized using a laser scanning confocal microscope. Only one oocyte at a time in each arm of the gonad matures prior to fertilization, approximately every 23 min. The mature oocyte becomes rounded and transits to the spermatheca, where the oocyte is immediately fertilized. The fertilized oocyte then exits the spermatheca and enters the uterus. Recordings of day 1 adult worms were initiated when rounding of the −1 oocyte was observed, which is a hallmark of meiotic maturation. A burst of fluorescence was detected immediately upon oocyte entry into the spermatheca (Fig. 2A; Movie 1). The first frame with visible fluorescence signal was designated as time 0. Notably, the calcium signal was apparent before ovulation into the spermatheca was completed, indicating that the meiotically mature −1 oocyte is in a fertilization-competent state before ovulation.

In agreement with previously reported work, the calcium wave illuminated by the CaFE reporter is biphasic (Samuel et al., 2001; Takayama and Onami, 2016). An initial burst phase localized to the site of sperm entry was followed by a slower calcium signal that traveled throughout the oocyte. The slow calcium wave terminated at the opposite pole from sperm entry and filled the entire oocyte for several minutes. The burst phase was complete by ∼5 s, whereas the slower wave phase peaked at ∼60 s. Unlike other species such as mice and humans, oscillations in the calcium signal were not observed.

Quantitation over seven independent replicates revealed an average increase in the signal of over 200% (Fig. 2B). Therefore, a robust signal was readily observable, even though the reporter was present as a single copy. The original dye-injection method reported a ∼30% increase in relative fluorescence (Samuel et al., 2001). Therefore, the CaFE reporter reflects the known kinetics of the calcium response during fertilization but is genetically encoded, has a greater signal-to-noise ratio and is easier to use.

### CaFE fluorescence is dependent on functional sperm

Fusion between the sperm and oocyte is required for fertilization. The calcium wave, as visualized using microinjected calcium-sensitive dyes, was observed only upon sperm fusion, and not upon sperm docking (Takayama and Onami, 2016). To test whether our CaFE reporter was detecting the fertilization-dependent calcium wave and not an illicit increase in fluorescence that coincides with ovulation, we examined the CaFE reporter in the background of the temperature-sensitive *spe9(hc88)* mutant (L'Hernault et al., 1988). Spe9 is a membrane protein specific to sperm and spermatids. At the restrictive temperature of 25°C, the *spe9(hc88)* mutant is unable to physically fuse with the oocyte (Singson et al., 1999). However, the *spe9(hc88)* mutant is still able to produce and release Major Sperm Protein (MSP), which is required for meiotic maturation and ovulation into the spermatheca.

**Fig. 3.**
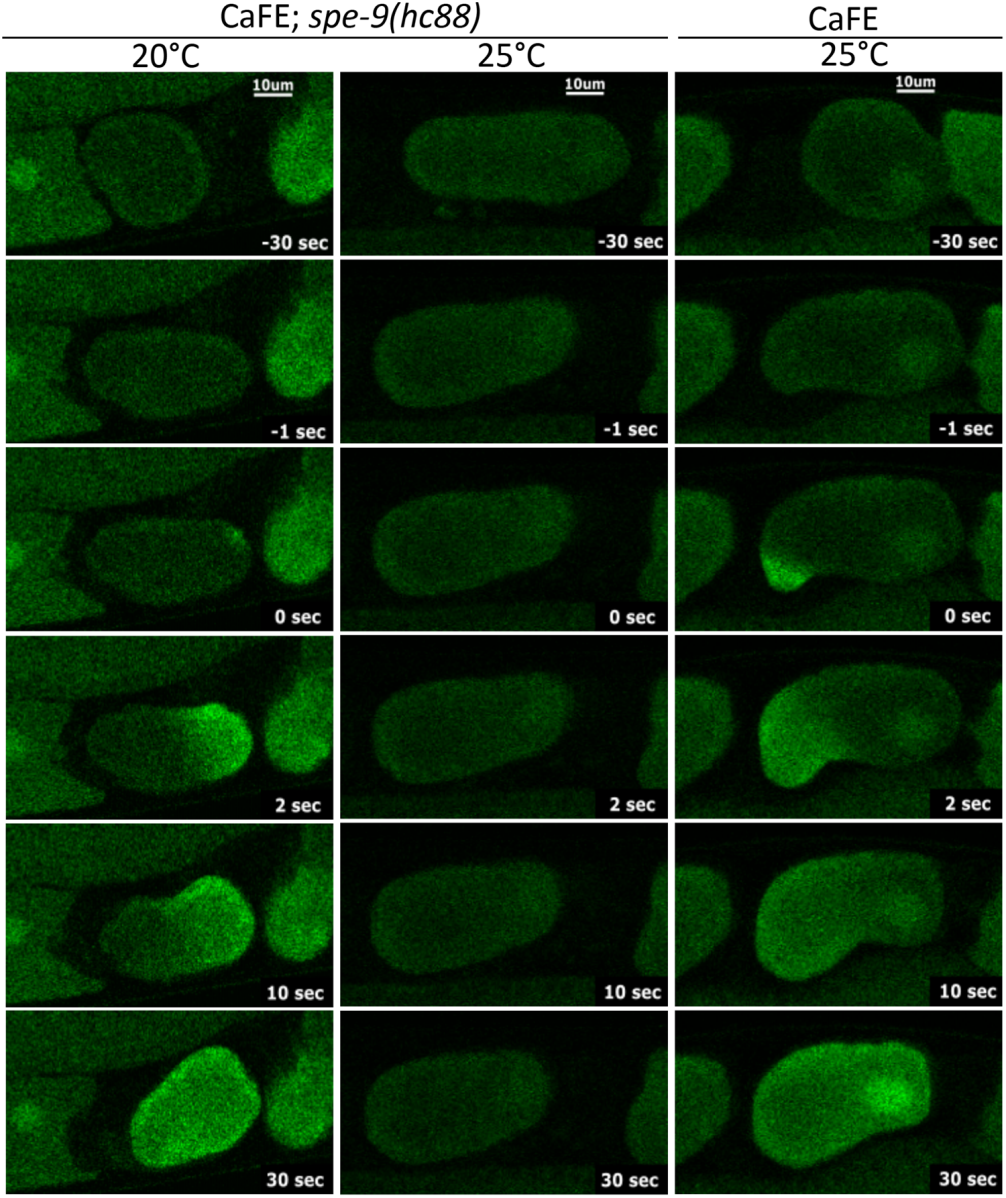
**CaFE fluorescence is dependent upon functional sperm.** Time-lapse images of ovulation in the EAG26 [CaFE;*spe-9(hc88)*] strain at the permissive temperature of 20°C (left column), the restrictive temperature of 25°C (middle column) or the control EAG16 (CaFE) strain at 25°C (right column). Scale bars: 10 μm Note that typical calcium waves associated with fertilization were observed at the permissive temperature in EAG26 and at the restrictive temperature for the control EAG16 strain.

We created a strain containing both the CaFE reporter and the *spe-9(hc88)* mutation. Worms were synchronized by a 2-h egg-lay window and grown at 20°C. Both CaFE and CaFE;*spe-9(hc88)* were shifted to the restrictive temperature of 25°C before the molt to L4 and then observed on a confocal microscope at day 1 or 2 of adulthood. No increase in fluorescence was observed at the restrictive temperature upon ovulation into the spermatheca in the CaFE;*spe-9(hc88)* strain, indicating that the fluorescence signal correlated with the ability to fertilize (Fig. 3, middle column). In contrast, the higher temperature had no significant effect on the fluorescence in the parent strain, indicating that the CaFE reporter was functional at 25°C (Fig. 3, right column). The CaFE reporter was functional in the *spe-9(hc88)* background as evidenced by the clear calcium wave visualized at the permissive temperature (Fig. 3, left column). Therefore, the fluorescence signal observed during ovulation by the CaFE reporter is dependent upon sperm fusion events during fertilization.

### Polyspermy events elicit an additional calcium signal

**Fig. 4.**
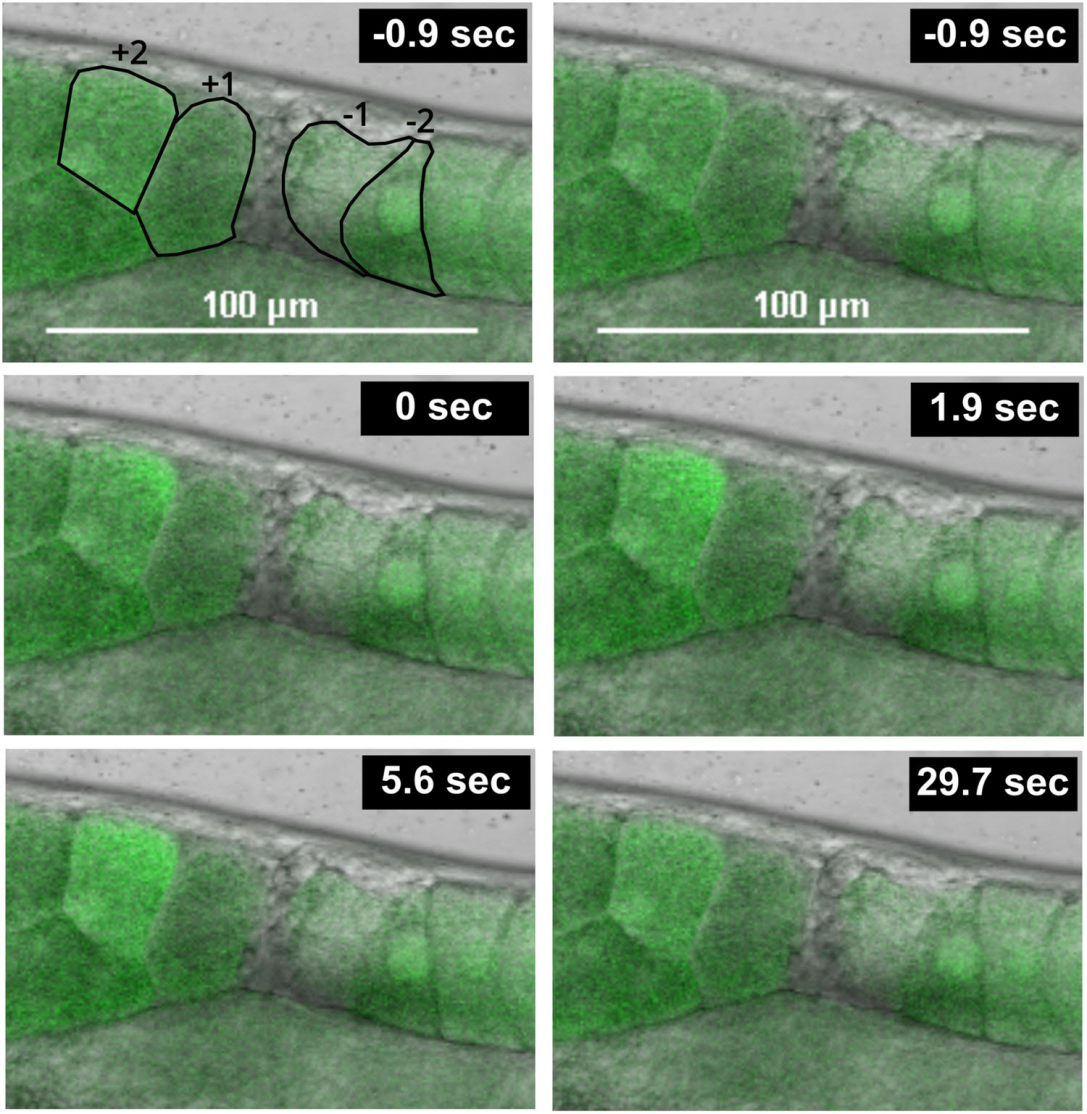
**The CaFE reporter reveals a late calcium wave during polyspermy.** Time-lapse images of a late calcium wave in the uterus of a worm treated with RNAi against *egg-3.* Time 0 was defined as the first frame exhibiting a signal in the +2 embryo. The positions of the −1 oocyte and the +1 and +2 embryos are marked in the first frame. Scale bars: 100 µm.

We next used the CaFE reporter to determine whether a polyspermy event would trigger an additional calcium wave. The eggshell provides an important barrier to polyspermy (Johnston et al., 2010). *egg-3* encodes a tyrosine phosphatase-like protein located on the oocyte plasma membrane and is essential for chitin eggshell formation. Defects in egg-3 have been reported to induce polyspermy (Maruyama et al., 2007). Embryos in the uterus of worms treated with RNAi knockdown of *egg-3* lacked the typical ovoid shape of healthy embryos and phenocopied eggs with defective eggshell formation (Fig. 4).

In *C. elegans,* oocytes typically display calcium transients only within the spermatheca. However, sperm are not restricted to the spermatheca and are routinely pushed out into the uterus during the transit of the oocyte. The amoeboid sperm then crawl back to the spermatheca. Previously, polyspermy events have been shown to occur in the uterus (Johnston et al., 2010).

We found that worms treated with *egg-3* knockdown had a clear calcium signal within the uterus, indicative of a polyspermy event (Fig. 4; Movie 2). Interestingly, the calcium wave during polyspermy also displayed similar dynamics as a fertilization event in the spermatheca. The uterine wave exhibited a biphasic nature, initiating at a single point on the cell surface and eventually filling the cell completely in a slow wave. These results indicate that the CaFE reporter can be used as an additional tool to study polyspermy.

### Summary

We established a fluorescent reporter system applied to early embryogenesis that marries a superior calcium-imaging tool combined with a model system with superlative genetics and a well-ordered gonad. This reporter reflects the known dynamics of the calcium response during fertilization but is genetically encoded, has a robust signal-to-noise ratio and is easy to use. Our results, utilizing polyspermy induced by RNAi, illustrate the utility of having a direct visual readout of fertilization in a genetically tractable and optically clear model organism. With the panoply of genetic tools and extensive RNAi library available in *C. elegans,* our CaFE reporter allows an unprecedented opportunity to dissect the OET.

## MATERIALS AND METHODS

### Strain construction

Nematodes were maintained using standard laboratory techniques on nematode growth medium (NGM) plates seeded with OP50 bacteria and incubated at 20°C unless otherwise indicated (Brenner, 1974). The N2 and PX696 strain was obtained through the *Caenorhabditis* Genetics Center (CGC). The jGCaMP7s sequence was based on the sequence of the pGP-CMV-jGCaMP7s plasmid (Addgene, #104463). This sequence was codon optimized for *C. elegans* and expressed behind the *spn-4* promoter and in front of the *pie-1* 3′ UTR. Three artificial introns were inserted into the coding sequence to promote expression. The reporter was synthesized and microinjected with a vector encoding the CRISPR protein and guide RNA to the *dpy-10* safe harbor locus by InVivo Biosystems. InVivo Biosystems also screened the microinjected lines and confirmed integration into the correct locus with PCR. The final strain (EAG16 *spn-4p::jGCaMP7s::pie-1u*) was confirmed to be hygromycin resistant, indicating correct integration in the safe harbor locus. The target locus for CRISPR integration is chromosome II:8420188-9420210.

The BA671 *spe-9(hc88)* strain was obtained through the CGC and crossed to the EAG16 to generate EAG26 (CaFE; *spe-9(hc88)*).

### Microscopy

Day 1 adults were anesthetized in 0.5 mM levamisole on a 3% agarose pad as previously described (Golden et al., 2020). Worms were imaged using a Nikon C2 laser scanning confocal microscope with Nomarski optics and a 20× air objective. Movies taken with a pinhole of 60 µm had a typical 488 nm laser power setting of 10. Movies taken with a pinhole of 30 µm had a typical 488 nm laser power setting of 41. Gain was set at ∼70 for all movies. All movies were collected at 1024×256 pixels. Videos were taken starting when the −1 oocyte showed signs characteristic of meiotic maturation, e.g. nuclear envelope breakdown and cortical cytoskeletal reorganization. Movies were collected as fast as possible with both Nomarski and fluorescence imaging at ∼0.91 s per frame. Typically, one movie was taken for quantitation per worm per recording session. Worms were exposed to levamisole for no more than 1 h.

### Image analysis

The Nikon NIS Elements Acquisition and Analysis software package was used to quantify changes in signal. The area encompassing the −1 oocyte and its path through the spermatheca was defined as the region of interest (ROI). The frame showing the first increase in fluorescence in the oocyte was defined as the 0 timepoint. The 15th frame preceding time 0 was taken as *F*_0_ or the baseline signal. The signal in the ROI was defined as (*F*_1_-*F*_0_)/*F*_0_. No corrections for signal decay were taken.

### RNAi

RNA interference was performed against *egg-3* with L4440 as the vector control. Feeding RNAi from the Ahringer library in *Escherichia coli* HT115(DE3) was used and expression was induced with 1 mM IPTG (Kamath and Ahringer, 2003). RNAi constructs were sequence verified. Worms were bleach-synchronized and exposed to RNAi starting from the L1 stage.

## Supplementary Material

10.1242/biolopen.059832_sup1 Supplementary information
