# Supplementary figures and images for: Visualization of the biphasic calcium wave during fertilization in Caenorhabditis elegans using a genetically encoded calcium indicator
Source: Biol Open. 2023 Aug 21;12(9):bio059832. doi: 10.1242/bio.059832 (PMC10655868; doi:10.1242/bio.059832)

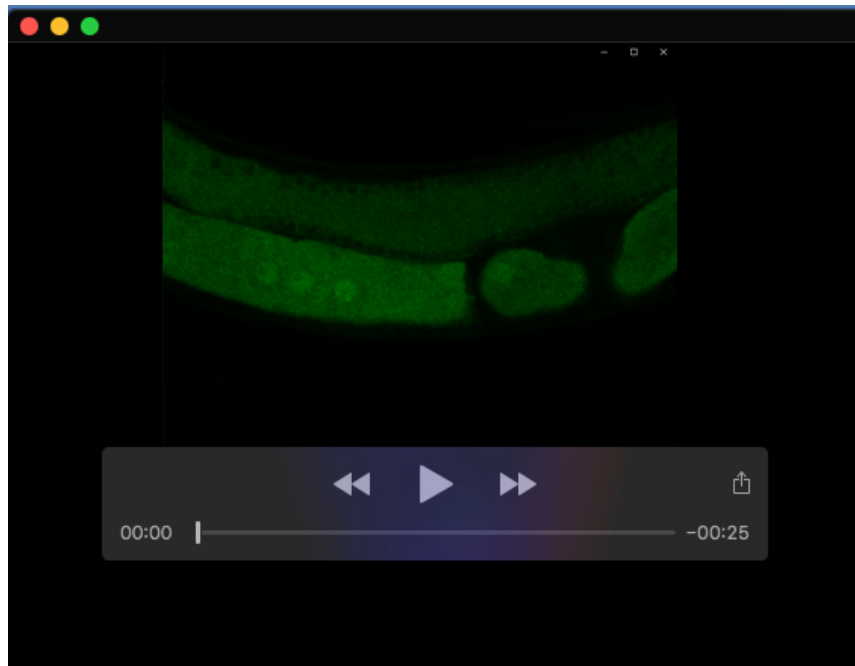

**Movie 1. Biphase calcium wave**

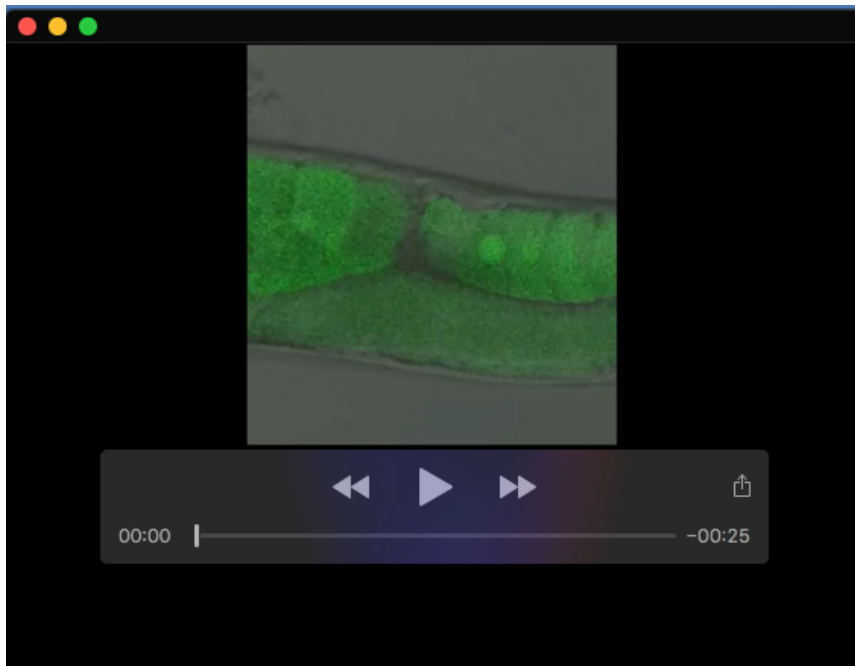

**Movie 2. Polyspermy**

Supplement: Supplementary information [file biolopen-12-059832-s1.pdf]
